# Supplementary material for: Supercurrent mediated by helical edge modes in bilayer graphene
Source: Nat Commun. 2024 Jan 29;15:856. doi: 10.1038/s41467-024-44952-6 (PMC10824753; doi:10.1038/s41467-024-44952-6)
Supplement: Supplementary file 1 — Supplementary Information [file 41467_2024_44952_MOESM1_ESM.pdf]

Supplementary information for "Supercurrent mediated by helical edge modes in bilayer graphene"

Prasanna Rout,[1] Nikos Papadopoulos,[1] Fernando Peñaranda,[2] Kenji Watanabe,[3] Takashi Taniguchi,[4] Elsa Prada,[2] Pablo San-Jose,[2] and Srijit Goswami[1]

[1]*QuTech and Kavli Institute of Nanoscience, Delft University of Technology, 2600 GA Delft, The Netherlands*

[2]*Instituto de Ciencia de Materiales de Madrid (ICMM), CSIC. Sor Juana Inés de la Cruz 3, 28049 Madrid, Spain*

[3]*Research Center for Functional Materials, National Institute for Materials Science, Tsukuba 305-0044, Japan*

[4]*International Center for Materials Nanoarchitectonics, National Institute for Materials Science, Tsukuba 305-0044, Japan*

CONTENTS

1. Device details 3

2. WSe_2/BLG/WSe_2 device: Dev A 3
 A. Gate map 3
 B. Arrhenius analysis 3
 C. Magnetoresistance 5

3. WSe_2/BLG/WSe_2 device: Dev B 6
 A. Gate map 6
 B. Fabry-Perot interference 7
 C. SQI pattern at high doping 7

4. Bare BLG device: Dev C 8

5. WSe_2/BLG/WSe_2 device: Dev D 8

6. Sheet resistance in IGP 9

7. SQI patterns for $n = 0$ as a function of D for Dev B, C and D 10

8. Formula for the critical current 11

9. Construction of the four-site minimal model 12

10. Regimes of the four-site model 14

11. Contribution to $I_c(\Phi)$ of $\mathcal{O}[\Delta^2\tau^2]$ processes 16

12. Construction of the multi-mode tight-binding model 18

13. Even-odd effect: Simple model 19

References 20

1. DEVICE DETAILS

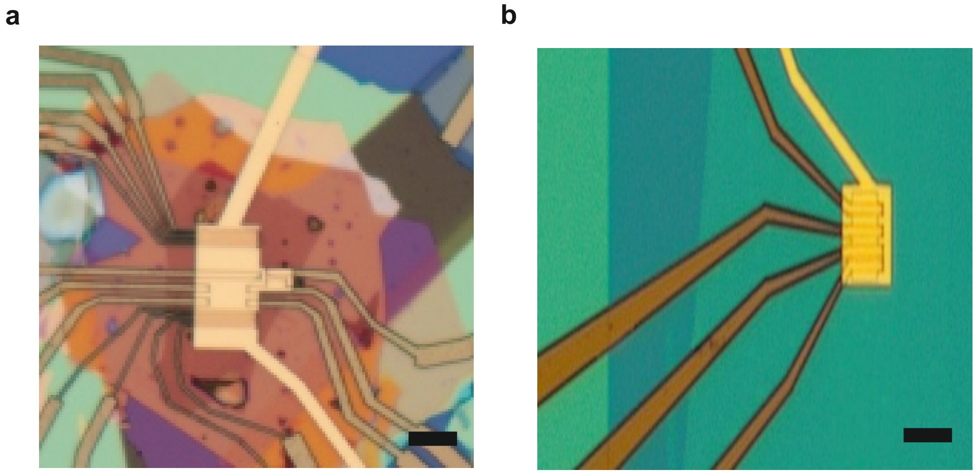

FIG. S1. Optical image of JJs fabricated on (a) hBN/WSe_2/BLG/WSe_2/hBN/graphite and (b) hBN/BLG/hBN/graphite stacks. Scale bar is 5 μm.

In this work we measure four devices: three JJs with the hBN/WSe_2/BLG/WSe_2/hBN stack (Dev A, Dev B, Dev D) and one JJ with hBN/BLG/hBN (Dev C). The two former JJs are 7 μm wide and their respective lengths are 3.7 μm (Dev A), 300 nm (Dev B) and 400 nm (Dev D). Dev C is 3 μm wide and 300 nm long.

2. WSe_2/BLG/WSe_2 DEVICE: DEV A

A. Gate map

Figure S2a shows the dual gate (back gate V_{BG} and top gate V_{TG}) map of the normal-state resistance R for Dev A. In Fig. S2b, R is replotted as a function of n and D. This clearly shows a resistance maximum near $n = 0 = D$ indicating the inverted-gap phase (IGP).

B. Arrhenius analysis

The Arrehenius analysis of the temperature dependence resistance is one of the widely used methods to determine the band gap of a homogeneous system. Our samples have

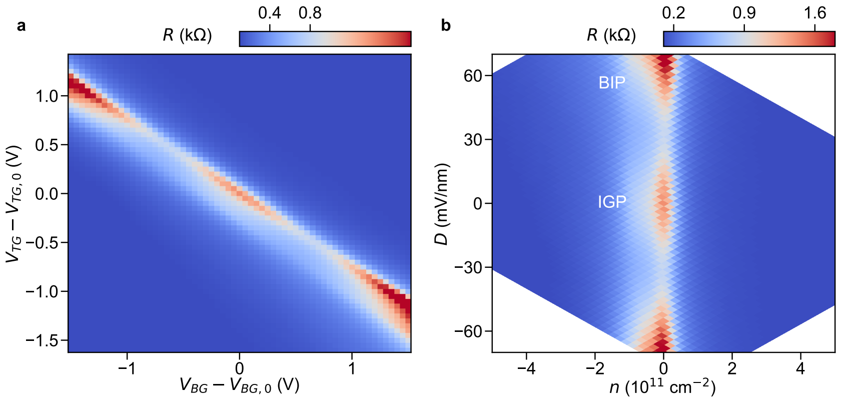

FIG. S2. (a) Normal-state resistance R measured as a function of V_{BG} and V_{TG} at 3.3 K for Dev A. Here $V_{BG,0} = 0.5$ V and $V_{TG,0} = -1.4$ V. (b) R replotted as a function of n and D using the capacitances $C_{BG} = 4.42 \times 10^{-4}$ F and $C_{TG} = 5.765 \times 10^{-4}$ F.

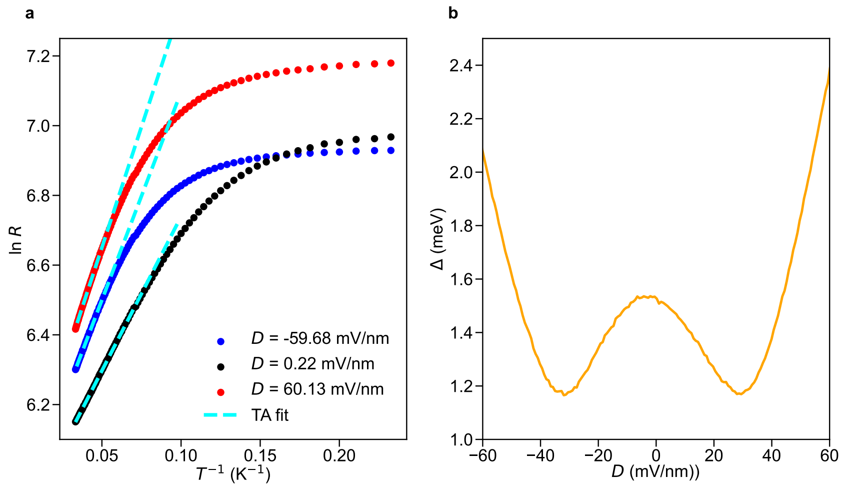

FIG. S3. (a) Arrhenius plot of the resistance for three different D. The linear fits to high temperature data indicates the thermal activatied (TA) conduction. (b) The gap Δ as a function of D extracted from TA model.

insulating bulk with parallel conduction channels as discussed in main text. Still we went ahead to perform a simplistic Arrehenius fit of our data [Fig. 2b in main text] to estimate the band gap for different D values. Figure S3a presents $\ln(R)$ as a function of T^{-1} for various D. The high temperature data show a linear dependence as expected for a thermal

activation model: $lnR \propto \Delta/k_BT$, where Δ is the band gap. The gap estracted from a fit to this formula shows a gap maximum around $D = 0$ as well as increasing band gap with increasing D within the BIP. The presence of these two gaps can be understood from the band structure presented in Fig. 1c (and the discussion in main text). Two local minima for Δ are observed at D = -32 and 29 mV/nm, which correspond to the inversion points, which gives a SOC coupling of $\lambda_I = 2.5$ and 2.2 meV.

C. Magnetoresistance

Figure S4a presents the resistance $R(D)$ at different in-plane magnetic fields B_y for Dev A while keeping $n = 0$. We observe a local maximum in R at $D = 0$ indicating the presence of band inversion for all B_y. We convert R to magnetoresistance MR $= [R(B_y) - R(0.3\ \mathrm{T})]/R(0.3\ \mathrm{T})$ in Fig. S4b (and also Fig. 2c in the main text).

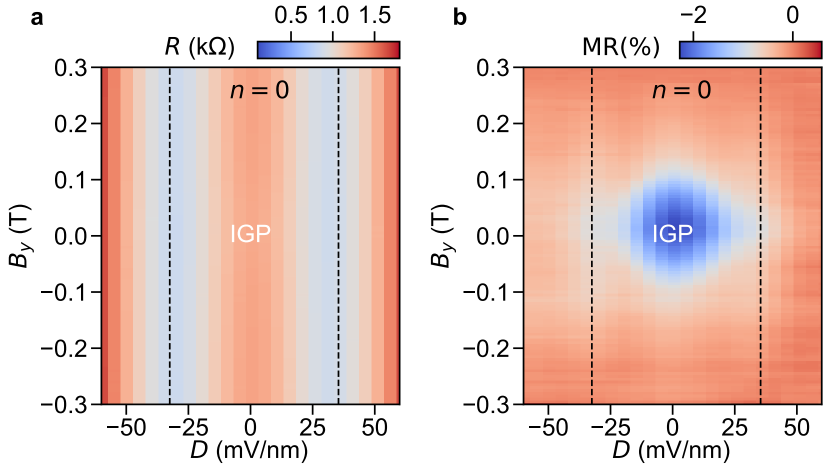

FIG. S4. (a) The resistance R and (b) magnetoresistance MR $= [R(B_y) - R(0.3\ \mathrm{T})]/R(0.3\ \mathrm{T})$ measured at 40 mK as a function of D and in-plane magnetic field B_y for Dev A. The dotted lines represent the inversion points.

3. WSe_2/BLG/WSe_2 DEVICE: DEV B

A. Gate map

The dual gate (back gate V_{BG} and top gate V_{TG}) map of the normal-state resistance R for Dev B is presented in Fig. S2a. Fig. S2b shows R replotted as a function of n and D, which reveals inverted-gap phase near $n = 0 = D$.

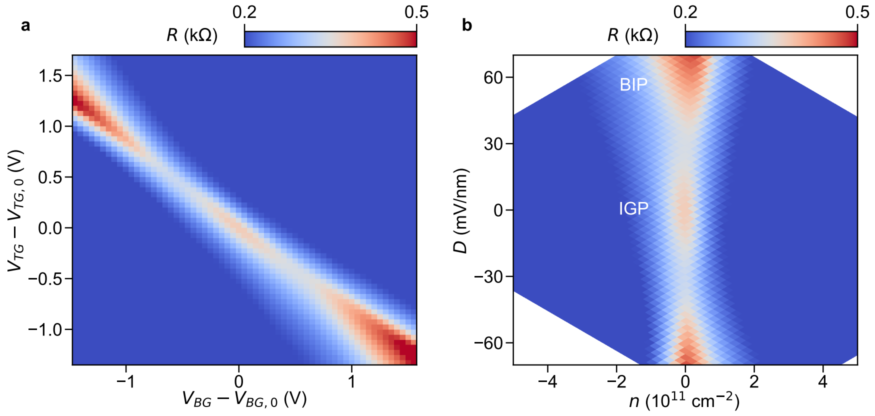

FIG. S5. (a) R measured as a function of V_{BG} and V_{TG} at 3.3 K for Dev B. Here $V_{BG,0} = 0.5$ V and $V_{TG,0} = -1.675$ V. (b) R replotted as a function of n and D using the capacitances $C_{BG} = 4.42 \times 10^{-4}$ F and $C_{TG} = 5.765 \times 10^{-4}$ F.

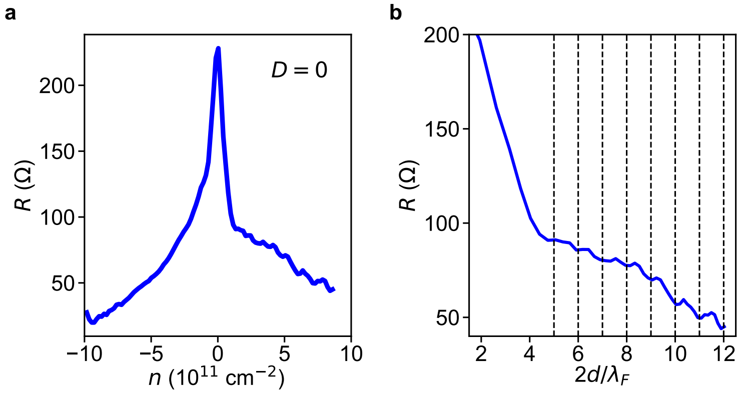

FIG. S6. (a) R measured as a function of carrier density n at displacement field $D = 0$ for Dev C. (b) R replotted as a function of $2L/\lambda_F$. The vertical dashed lines mark the period of the Fabry-Perot oscillations.

B. Fabry-Perot interference

The existence of Fabry-Perot (FP) interferences in the normal-state resistance curve shows that Dev B is a ballistic JJ (Fig. S6). The FP resonances appear when the condition $2d = m\lambda_F$ is satisfied, where d is the cavity length, m is an integer and λ_F is the Fermi wavelength. Figure S6b shows the resistance oscillations with a period of $2d/\lambda_F$ for $d =$ 325 nm.

C. SQI pattern at high doping

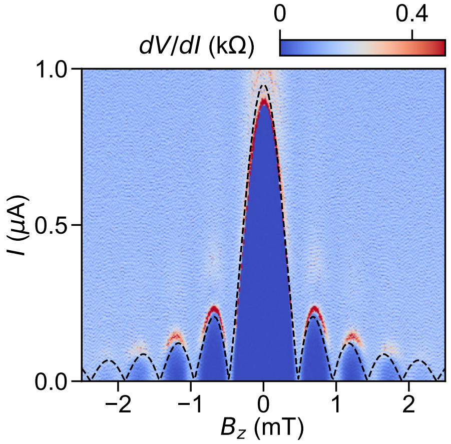

FIG. S7. Superconducting quantum interference pattern $I_c(B_z)$ for Dev B at $n = 3 \times 10^{11}$ cm^{-2} and $D = 0$, that follows the standard Fraunhofer curve (dashed line).

Figure S7 displays a Fraunhofer superconducting quantum interferometry (SQI) pattern for Dev B at $n = 3\times10^{11}$ cm^{-2} and $D = 0$. The SQI for a JJ with homogeneous supercurrent flow is of the form: $I_c(B_z) = I_{c,0}\sin(\pi\Phi/\Phi_0)/(\pi\Phi/\Phi_0)$, where $I_{c,0}$ is the zero-field critical current and $\Phi_0 = h/2e$ is the (superconducting) flux quantum. The magnetic flux Φ passing through the JJ is given by B_z/LW, where L and W are the length and width of the junction, respectively. However, for a practical junction, the superconducting contacts expel flux, which leads to enhanced field in the junction region[1–3]. This flux focusing effect leads to the Fraunhofer pattern varying more rapidly with magnetic field, and therefore the effective area (or length) increases. For our JJs the magnetic penetration depth of NbTiN ($\approx$ 400 nm)[4] is

larger than the film thickness (≈110 nm) and the width of the electrode (l ≈300 nm). Thus, the effective JJ length can be estimated as: $L + l = 600$ nm[3]. Using this value we plot the standard Fraunhofer curve in Fig. S7, which matches quite well the measured SQI pattern.

4. BARE BLG DEVICE: DEV C

Figure S8 shows the resistance R as a function of V_{BG} and V_{TG} (a) as well as a function of n and D (b) of the BLG JJ without any WSe_2 encapsulation. We observe an increase in R with increasing $|D|$ due to the opening of a trivial gap (Fig. S8c). In addition, the critical current I_c gradually reduces with increasing $|D|$ as expected from the $R(D)$ behaviour.

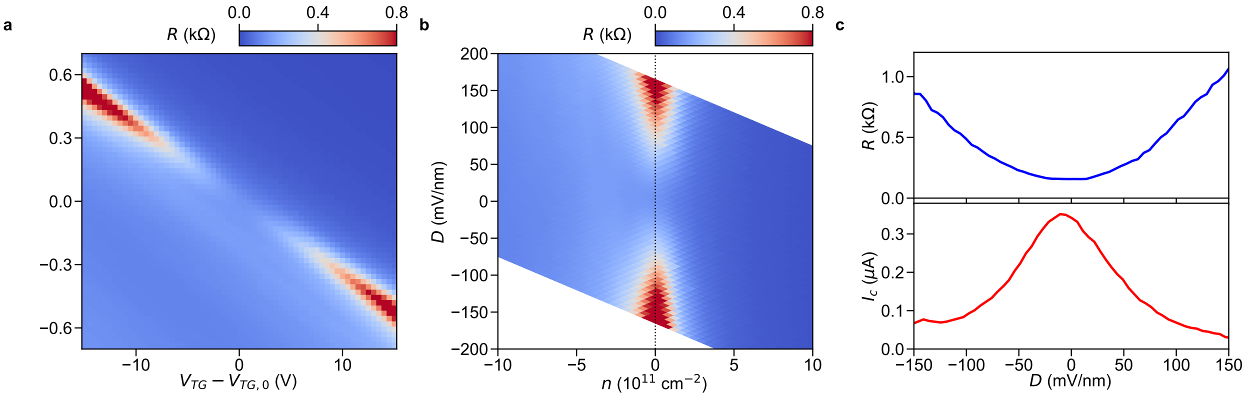

FIG. S8. (a) R measured as a function of V_{BG} and V_{TG} at 3.3 K for Dev C. Here $V_{BG,0} = 0$ V and $V_{TG,0} = -0.07$ V.(b) R replotted as a function of n and D using the capacitances $C_{BG} = 0.96 \times 10^{-4}$ F and $C_{TG} = 2.75 \times 10^{-3}$ F. (c) Resistance line cut $R(D)$ of (b) at $n = 0$ (top panel) and critical current $I_c(D)$ for $n = 0$ at 40 mK (bottom panel).

5. WSe_2/BLG/WSe_2 DEVICE: DEV D

We show the dual gate (back gate V_{BG} and top gate V_{TG}) map of the resistance R for Dev D in Fig. S9a and replot R as a function of n and D in Fig, S9b. This shows a resistance maximum near $n = 0 = D$ indicating the inverted-gap phase.

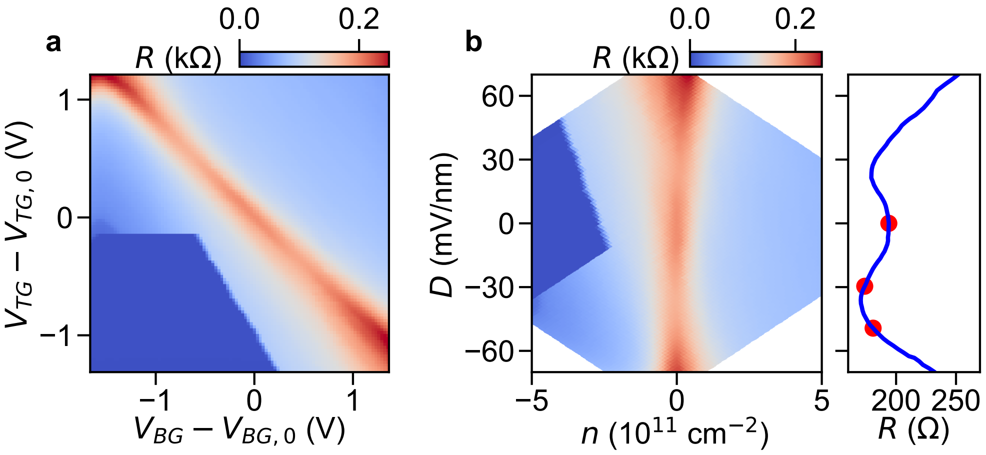

FIG. S9. (a) The resistance R measured as a function of V_{BG} and V_{TG} at 1.4 K for Dev D. Here $V_{BG,0} = 0.15$ V and $V_{TG,0} = -0.2$ V. (b) R replotted as a function of n and D using the capacitances $C_{BG} = 4.9 \times 10^{-4}$ F and $C_{TG} = 5.765 \times 10^{-4}$ F.

6. SHEET RESISTANCE IN IGP

Figure S10 presents the sheet resistance R_s measured at $D = 0 = n$ for Dev A, B and D, which is given by the relation: $R_s = RW/L$. Here L and W are the length and width of the device mentioned in the section 1. The resistance R values at $D = 0 = n$ are determined from the dual gate maps presented in Fig. S2, S5, and S9.

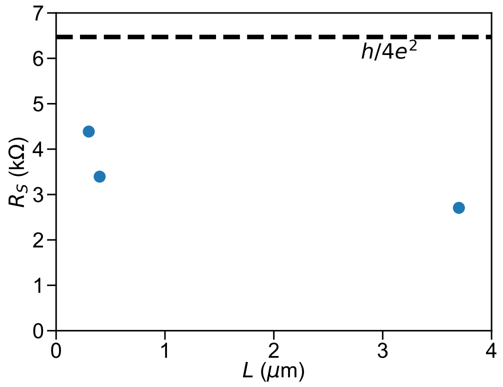

FIG. S10. The sheet resistance R_S measured at $D = 0 = n$ for Dev A, B and D. The dotted lines represent the theoretically predicted value of $h/4e^2$.

7. SQI PATTERNS FOR $n = 0$ AS A FUNCTION OF D FOR DEV B, C AND D

Figure S11 presents the SQI patterns measured at larger D values and B_z for Dev B and Dev C. As discussed in main text, the even-odd effect is only seen in the WSe_2-encapsulated JJ. For large D values we observe symmetric SQUID-like SQI patterns for both JJs indicating the presence of trivial edge states. These edge states have been previously observed in BLG JJs[5,6].

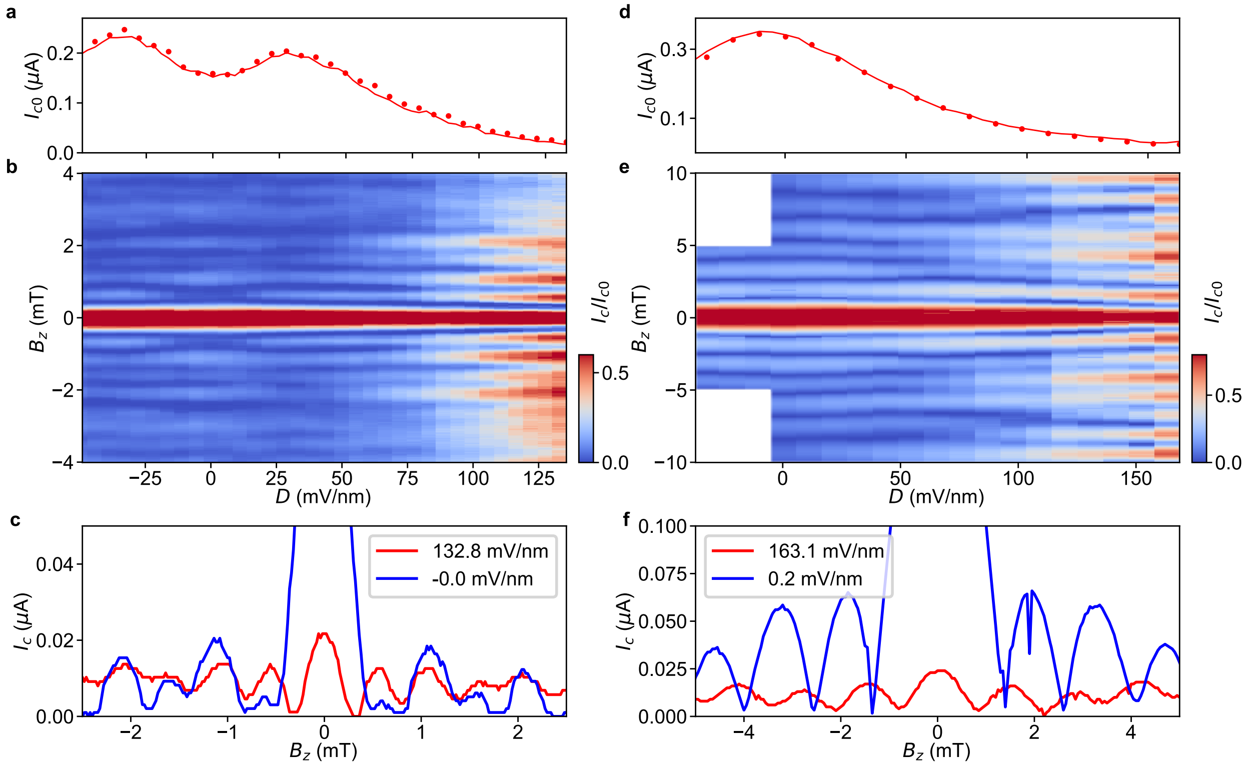

FIG. S11. (a) Zero-field critical current I_{c0} extracted from Fig. 3(b) of the main text (solid line) and a line cut at $B_z = 0$ of (b) (solid circles) as a function of D for Dev B. (b) SQI pattern as a function of B_z and D at $n = 0$ for Dev B. (c) Line cuts of (b) at $D = 0$ mV/mm and $D = 132.8$ mV/mm. (d) Zero-field critical current I_{c0} extracted from Fig. S8(c) (solid line) and a line cut at $B_z = 0$ of (e) (solid circles) as a function of D for Dev C. (e) SQI pattern as a function of B_z and D at $n = 0$ for Dev C. (f) Line cuts of (e) at $D = 0.2$ mV/mm and $D = 163.1$ mV/mm.

Figure S12 presents the SQI patterns measured at three different D values for Dev D. As observed in Dev B, the even-odd effect is clearly observed for $D = -1$ mV/mm (IGP). At D =49 mV/mm, we do not see this effect and the intensities of side lobes gradually

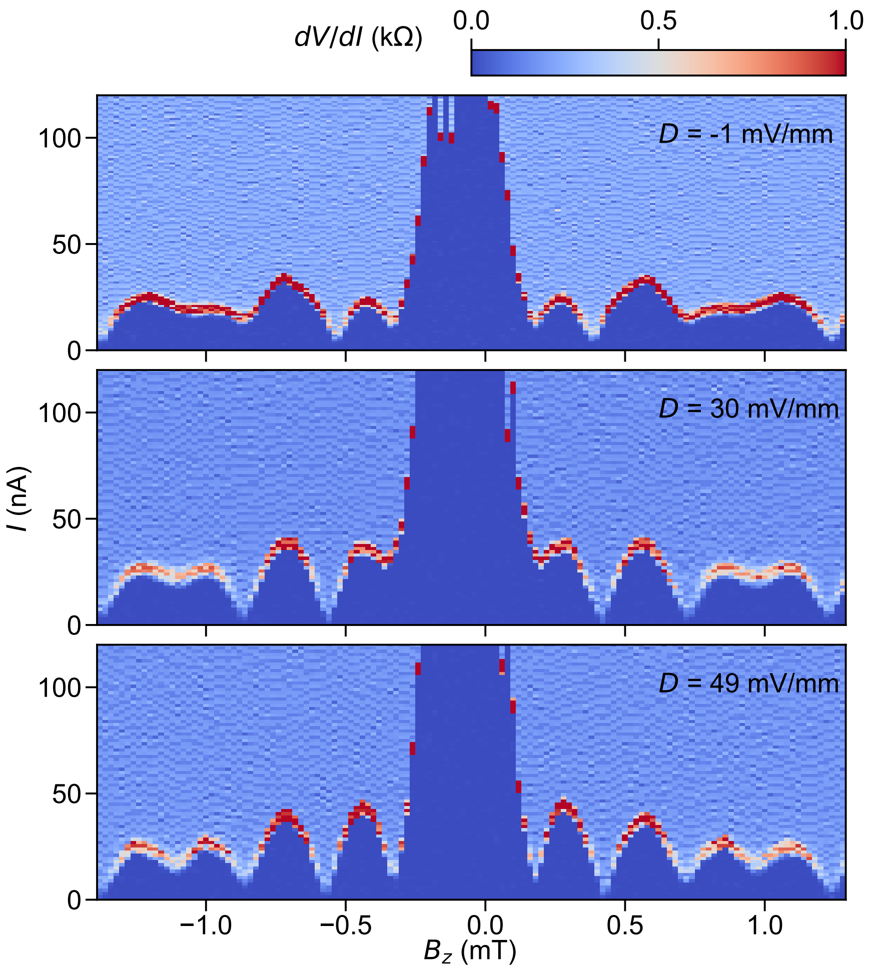

FIG. S12. SQI patterns measures at $D = -1$ mV/mm (upper panel), 30 mV/mm (middle panel), and 49 mV/mm (lower panel). These three D values are marked as red circle in Fig. S9b.

decreases.

8. FORMULA FOR THE CRITICAL CURRENT

Neglecting phase fluctuations, the critical current across a two-terminal JJ can be written as

$$I_c(\Phi) = \max_{2\pi \geq \phi \geq 0} \left| I(\phi, \Phi) \right|, \tag{1}$$

where $I(\phi, \Phi)$ is the supercurrent at a given superconducting phase difference, ϕ, and magnetic flux, Φ, threading the junction area. In the zero-temperature limit, $I(\phi, \Phi)$ can in turn be expressed purely as a function of spectral quantities, namely the system's free energy F,

as

$$I(\phi, \Phi) = \frac{2e}{\hbar} \frac{d}{d\phi} F(\phi, \Phi) = \frac{2e}{\hbar} \frac{d}{d\phi} \sum_{n \in \mathrm{occ}} E_n(\phi, \Phi), \tag{2}$$

written as the sum of the energies E_n over all occupied eigenstates of the junction Hamiltonian H. These correspond to negative eigenvalues of the Bogoliubov-de Gennes (BdG) Hamiltonian.

9. CONSTRUCTION OF THE FOUR-SITE MINIMAL MODEL

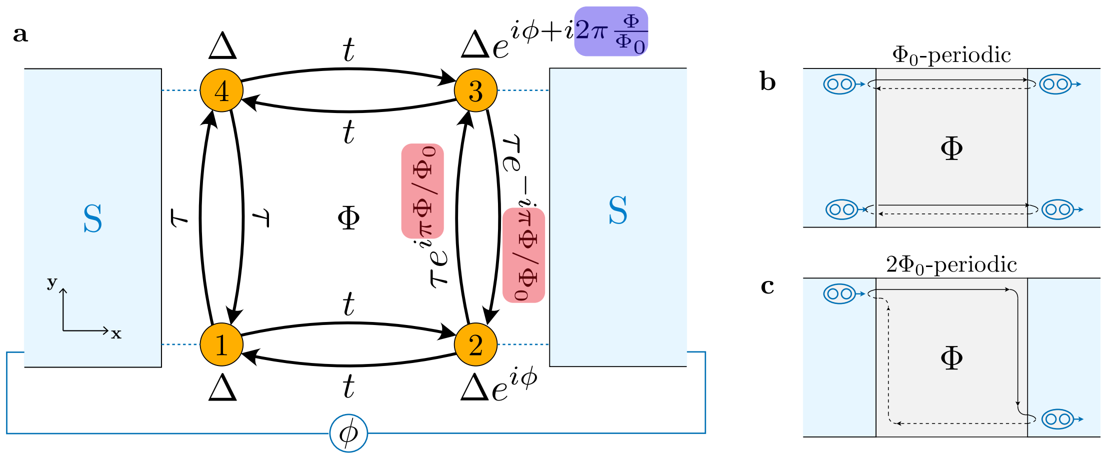

FIG. S13. **(a)** Sketch of a minimal model for the Josephson junction (JJ) with vacum edge states, defined by hoppings t, and an inter-edge coupling, defined by hoppings τ. Sites 1, 4 and 2, 3 are coupled to the left and right superconducting leads, respectively, which have a phase difference ϕ. In addition, a finite magnetic flux Φ induces Peierls and superconducting phases at the hopping amplitudes and onsite pairing terms, respectively. Crucially, the Peierls phase on those hoppings connecting different edges (in red) halves the flux-induced superconducting phase at site 3 (in blue). The induced superconducting pairing amplitudes at the corners are Δ. **(b)** Supercurrent trajectories that contribute to a Φ_0-periodic SQUID-like Fraunhofer pattern. **(c)** Trajectories encircling the normal region enabled by inter-edge coupling, that contribute with $2\Phi_0$-periodic critical currents.

In order to get physical insight into the processes that may give rise to an even-odd modulation in $I_c(\Phi)$, we propose, prior to more elaborate calculations, the following minimal tight-binding model for the junction. It consists of only four sites located at the corners

of the junction, see Fig. S13(a). These four sites represent the normal region between the superconductors, and are threaded by a magnetic flux Φ. Within this model, the sites labelled as 1 and 4, and 2 and 3 are contacted to the left and right superconducting leads, respectively, in a JJ geometry. The resulting BdG Hamiltonian for a single spin species reads

$$H = \begin{pmatrix} H_0 & H_\Delta^+ \\ H_\Delta & -H_0^* \end{pmatrix}, \tag{3}$$

written in the Nambu basis $(\boldsymbol{c}_\sigma, \boldsymbol{c}_{\bar{\sigma}}^\dagger)^T$ where $\boldsymbol{c}_\sigma = (c_{1\sigma}, c_{2\sigma}, c_{3\sigma}, c_{4\sigma})$ and

$$H_0 = \begin{pmatrix} 0 & t & 0 & \tau \\ t & 0 & \tau e^{-i\pi\Phi/\Phi_0} & 0 \\ 0 & \tau e^{i\pi\Phi/\Phi_0} & 0 & t \\ \tau & 0 & t & 0 \end{pmatrix}, \quad H_\Delta = \Delta \begin{pmatrix} 1 & 0 & 0 & 0 \\ 0 & e^{i\phi} & 0 & 0 \\ 0 & 0 & e^{i\phi+i2\pi\Phi/\Phi_0} & 0 \\ 0 & 0 & 0 & 1 \end{pmatrix}. \tag{4}$$

H_0 and H_Δ are 4×4 matrices corresponding to the normal Hamiltonian of the particle sector and the onsite superconducting electron-hole pairing terms induced by the leads, respectively. Δ and ϕ are the induced pairing amplitude and phase from the parent superconductors, respectively. t and τ are the hopping amplitudes between sites along the vacuum edge interfaces ($1 \leftrightarrow 2$ and $3 \leftrightarrow 4$) and the NS interfaces ($1 \leftrightarrow 4$ and $2 \leftrightarrow 3$), respectively. Thus, the ratio τ/t is a key quantity that controls the efficiency of the inter-edge coupling (between the top and bottom edges).

For the sake of simplicity, we assume that the system is spin degenerate and, thus, Eq. (3) can be regarded as half the BdG Hamiltonian of a spinful model. The previous assumption is justified as long as the magnetic length at $\Phi = \Phi_0$ is the largest spatial scale of the problem, which seems a good approximation for large-area devices.

The vector potential is chosen in the gauge $\mathbf{A} = A_y\mathbf{y} = \frac{\Phi}{LW}x\mathbf{y}$, where L and W are the length and width of the junction, respectively. The origin of coordinates is chosen at site 1. The magnetic flux introduces a position-dependent modulation of the pairing phase, following $\Delta_{\text{parent}}(\mathbf{r}) = \Delta_{\text{parent}}(\mathbf{r}_0)\exp(2i\int_{\mathbf{r}_0}^{\mathbf{r}}\mathbf{A}\cdot d\mathbf{r})$, where $\mathbf{r}_0$ is any given point inside the superconductor and the integral path is taken inside it. Taking $\mathbf{r}_0$ in the left lead, adjacent to site 1, this introduces a phase ϕ at site 2 (junction phase difference picked along the outer loop between left and right) and $\phi + 2\pi\Phi$ at site 3. The latter makes the Hamiltonian Φ_0-periodic in Φ. In addition, Φ enters H_0 as a Peierls phase in the hoppings. In the chosen

gauge, the Peierls phase is non-zero only for hoppings connecting sites $2 \leftrightarrow 3$. The Peierls phase makes H $2\Phi_0$-periodic in Φ, in contrast to the Φ_0-periodicity from the pairing phases. From a different point of view, electrons and holes will pick up an Aharonov phase $\pm\pi\Phi/\Phi_0$ when circulating around the normal region (possible only if $\tau \neq 0$, which enables inter-edge scattering), which will produce a beating when added to the $2\pi\Phi/\Phi_0$ phase of a standard SQUID discussed in the main text. Thus, and despite its extreme simplicity, the four-site model allows us to identify inter-edge scattering as the key mechanism behind the even-odd effect.

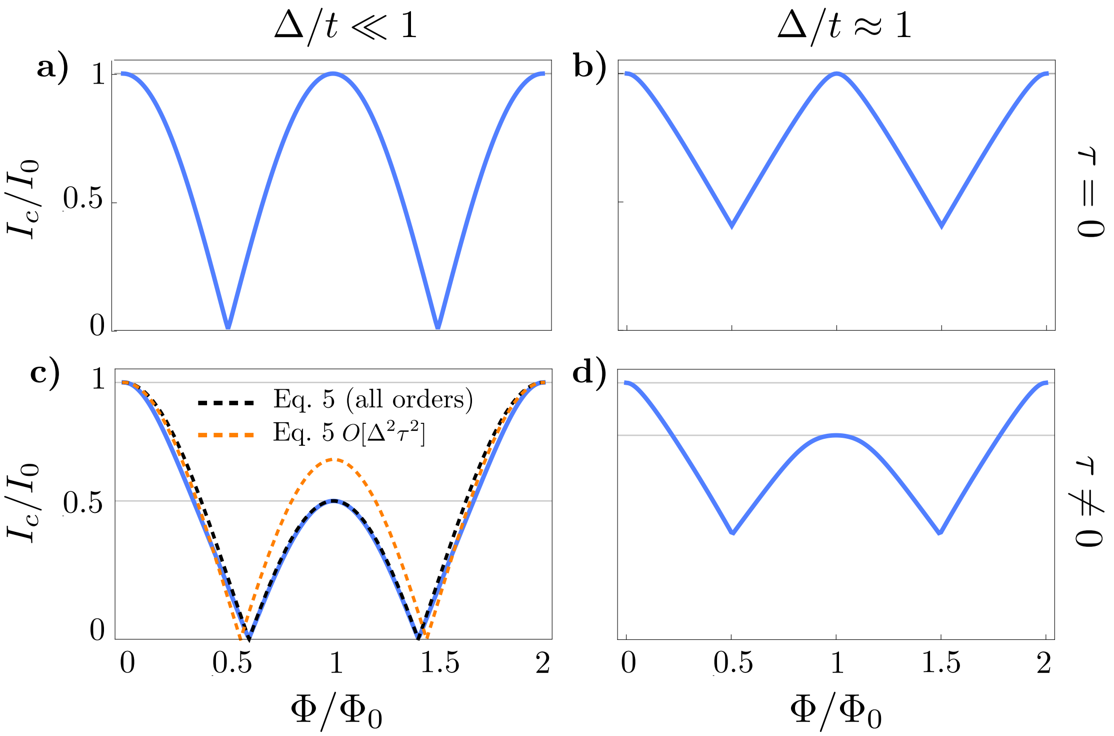

FIG. S14. Regimes of the minimal four-site model. $\Delta/t = 0.1$ (a, c), $\Delta/t = 0.9$ (b, d), $\tau/t = 0$ (a, b), $\tau/t = 0.5$ (c, d).

10. REGIMES OF THE FOUR-SITE MODEL

Here we compute the critical current $I_c(\Phi)$ using Eqs. (1) and (2) and the spectrum of Eq. (3) in four different regimes. The results are shown in Fig. S14. The left and right columns correspond to the $\Delta/t \ll 1$ and $\Delta/t \approx 1$ regimes, respectively, whereas the top and

bottom row contains the cases of forbidden or allowed inter-edge coupling controlled by τ/t. In the absence of inter-edge coupling, Fig. S14(a,b), the spectrum has Φ_0-periodicity whose oscillations with Φ may or may not touch zero at half-integer fluxes depending on the value of Δ/t. A small Δ/t ratio yields a complete suppression of I_c at half-integer normalized flux (a) as opposed to (b).

The beating of the Φ_0-periodic modulation comes from electron/hole trajectories encircling the sample [see Fig. 4(d, e) of the main text] and is enabled by a finite τ/t. In Fig. S14(c), the experimentally relevant situation, the exact $I_c(\Phi)$ functional form, in blue, can be approximated by

$$I_c(\Phi) \approx \frac{I_c(0) + I_c(\Phi_0)}{2I_c(0)} \left| \cos(\pi\frac{\Phi}{\Phi_0}) + \frac{I_c(0) - I_c(\Phi_0)}{I_c(0) + I_c(\Phi_0)} \right| , \tag{5}$$

depicted as a dashed black line, similar to Eq. (18) of Ref. 7 obtained in the framework of a network (transfer-matrix) model. This approximation becomes exact, however, only in the limit of small Δ/t and τ/t. In the general case, in contrast, the critical current receives contributions from all possible processes without spin mixing that lead to the net transfer of Cooper pairs across the junction and, therefore, involve an arbitrary number of loops around the sample and Andreev electron/hole conversions. To gain a better understanding of those that contribute to the Φ_0-periodicity breaking, we focus on the leading processes up to order $(\Delta/t)^2$ and $(\tau/t)^2$ corresponding to two Andreev reflections, one at each NS interface, and quasiparticle trajectories that encircles the device at most once, as those depicted in Fig. S13(b, c). In this $\Delta/t, \tau/t \ll 1$ limit, the critical currents at $\Phi = 0$ and $\Phi = \Phi_0$ read:

$$I_c(0) \approx \frac{2e}{\hbar}\frac{16\Delta^2}{t}\left(1 + \frac{\tau^2}{t^2}\right) , \tag{6}$$

and

$$I_c(\Phi_0) \approx \frac{2e}{\hbar}\frac{16\Delta^2}{t}\left(1 - \frac{\tau^2}{2t^2}\right) . \tag{7}$$

Substituting Eqs. (6) and (7) into Eq. (5) yields the dashed orange line in Fig. S14(c) that tends to the exact solution as $\tau/t \to 0$. Despite its deviation from the calculations to all orders (blue and dashed black lines in Fig. S14) as we relax the $\tau/t \ll 1$ condition, we conclude that loops of order $(\Delta/t)^2$ and $(\tau/t)^2$ are the most relevant within the hierarchy of inter-edge processes that leads to $2\Phi_0$-periodicity.

11. CONTRIBUTION TO $I_c(\Phi)$ OF $\mathcal{O}[\Delta^2\tau^2]$ PROCESSES

In this section we provide details of the derivation of Eqs. (6) and (7). We start by rewritting the BdG Hamiltonian of Eq. (3) into a normal and a superconducting part as:

$$H/t = \tilde{H}_N + \tilde{H}_S = \begin{pmatrix} H_0/t & 0 \\ 0 & -H_0^*/t \end{pmatrix} + \Delta/t \begin{pmatrix} 0 & H_\Delta^+/\Delta \\ H_\Delta/\Delta & 0 \end{pmatrix}. \quad (8)$$

In the regime of interest for the experiment, i.e., that of Fig. S14(c) with $\Delta/t \ll 1$, $\tilde{H}_S$ can be regarded as a small perturbation to $\tilde{H}_N$ and, therefore, a perturbative treatment of the problem is justified. Let $|\Psi_i^{(0)}\rangle$ be the i'th eigensolution of

$$\tilde{H}_N|\Psi_i^{(0)}\rangle = E_i^{(0)}|\Psi_i^{(0)}\rangle, \quad (9)$$

with energy $E_i^{(0)}$. Since the unperturbed Hamiltonian does not couple the electron and hole sectors we can distinguish said eigenstates by their electron/hole character:

$$|\Psi_{i,e}^{(0)}\rangle = |e\rangle \otimes |\psi_{i,e}^{(0)}\rangle, \quad (10)$$

$$|\Psi_{i,h}^{(0)}\rangle = |h\rangle \otimes |\psi_{i,h}^{(0)}\rangle, \quad (11)$$

where $|e\rangle$ and $|h\rangle$ are the eigenstates of the Nambu projector $\mathbb{P}_{e/h} = \tau_0 \pm \tau_z$, respectively, and $|\psi_{i,e}^{(0)}\rangle$ ($|\psi_{i,h}^{(0)}\rangle$) the eigenstates of H_0 ($-H_0^*$). They obey the so-called biorthonormality conditions, which in this basis reads:

$$\langle\Psi_{i,e}^{(0)}|\Psi_{j,e}^{(0)}\rangle = \delta_{i,j}, \quad \langle\Psi_{i,h}^{(0)}|\Psi_{j,h}^{(0)}\rangle = \delta_{i,j}, \text{ and } \langle\Psi_{i,h}^{(0)}|\Psi_{j,e}^{(0)}\rangle = 0, \quad (12)$$

and

$$\langle\psi_{i,e}^{(0)}|\psi_{j,e}^{(0)}\rangle = \langle\psi_{i,h}^{(0)}|\psi_{j,h}^{(0)}\rangle = \delta_{i,j}. \quad (13)$$

Under standard perturbation theory the eigenstates and eigenvalues of H can be expressed as:

$$E_i = E_i^{(0)} + E_i^{(1)} + E_i^{(2)} + \mathcal{O}[(\Delta/t)^3] \quad \text{and} \quad (14)$$

$$|\Psi_i\rangle = |\Psi_i^{(0)}\rangle + |\Psi_i^{(1)}\rangle + |\Psi_i^{(2)}\rangle + \mathcal{O}[(\Delta/t)^3]. \quad (15)$$

where $E_i^{(n)}$ and $|\Psi_i^{(n)}\rangle$ corresponds to the n'th order contribution to the i'th eigenstate $|\Psi_i\rangle$ with corresponding energy E_i of the perturbed problem, respectively.

Substituting the previous expansion into the Schrödinger equation for the full BdG Hamiltonian yields:

$$\left(H^{(0)} - E_i^{(0)}\right)|\Psi_i^{(1)}\rangle = \left(E_i^{(1)} - H^{(1)}\right)|\Psi_i^{(0)}\rangle, \tag{16}$$

and

$$\left(H^{(0)} - E_i^{(0)}\right)|\Psi_i^{(2)}\rangle = E_i^{(2)}|\Psi_i^{(0)}\rangle + \left(E_i^{(1)} - H^{(1)}\right)|\Psi_i^{(1)}\rangle. \tag{17}$$

The artificial built-in redundancy of the Nambu representation for $\tilde{H}_N$ demands to use degenerate perturbation theory, as it reveals in the form of a two-fold degeneracy of the unperturbed energy levels, eventually splitted by the perturbation $\tilde{H}_S$. Following Ref. 8 we apply the following ansatz for the i-th two-fold degenerate subspace of the unperturbed system:

$$|\Psi_i^{(0)}\rangle = \alpha_i|\Psi_{i,e}^{(0)}\rangle + \beta_i|\Psi_{i,h}^{(0)}\rangle, \tag{18}$$

where α and β are constants chosen to be real for simplicity. In a similar fashion, higher order eigenstates with $n > 0$ can be decomposed as:

$$|\Psi_i^{(n)}\rangle = \sum_{j\neq i} \alpha_j^{(n)}|\Psi_{j,e}^{(0)}\rangle + \beta_j^{(n)}|\Psi_{j,e}^{(0)}\rangle. \tag{19}$$

Note that the $i \neq j$ constraint in the sum together with Eq. (12) imposes that the first and subsequent perturbed eigenstates are orthogonal to $|\Psi_i^{(0)}\rangle$, being consistent with the normalisation choice: $\langle\Psi_i^{(0)}|\Psi_i\rangle = 1$.

The first and second energy corrections to $E_i^{(0)}$ can be readily found by substituting these ansatszes into Eq. (16) and Eq. (17), respectively, and solving the system of equations resulting from left multiplying it by $\langle\Psi_{i,e/h}^{(0)}|$. After some algebraic manipulations (see Ref. 8 for instance) we get: $E_i^{(1)} = 0$, since the BdG Hamiltonian is hermitian. On the other hand, the second-order corrections are given by:

$$E_{i,e}^{(2)} = \sum_{j\neq i} \frac{\langle\psi_{i,e}|\Delta|\psi_{j,h}\rangle\langle\psi_{j,h}|\Delta^*|\psi_{i,e}\rangle}{E_j^{(0)} - E_i^{(0)}}, \tag{20}$$

and similarly

$$E_{i,h}^{(2)} = \sum_{j\neq i} \frac{\langle\psi_{i,h}|\Delta^*|\psi_{j,e}\rangle\langle\psi_{j,e}|\Delta|\psi_{i,h}\rangle}{E_j^{(0)} - E_i^{(0)}}, \tag{21}$$

with $i, j \in \{1, 2, 3, 4\}$. Note that the bras and kets are the eigenstates of the normal 4×4 Hamiltonian H_0/t instead of those of the 8×8 BdG $\tilde{H}_N$ in Eq. (8), a circumstance that greatly reduces the algebraic complexity.

Now that we know the second order corrections to $E_i^{(0)}$, we can compute the free energy summing over all i's corresponding to occupied states, and by virtue of Eqs. (2) and (1) compute $I_c(\Phi)$. Taking the $\Phi \to 0$ and $\Phi \to \Phi_0$ limits of the resulting expression yields Eqs. (6) and (7).

12. CONSTRUCTION OF THE MULTI-MODE TIGHT-BINDING MODEL

Going beyond the four-site model, we consider the following tight-binding Hamiltonian

$$H_{TB} = H_{\rm regions} + H_{\rm coupling}, \tag{22}$$

where $H_{\rm regions}$ comprises the Hamiltonians of three different regions [see sketch in Fig. 4(c) of the main text] and $H_{\rm coupling}$ the couplings between them. The former reads

$$H_{\rm regions} = H_{\rm IGP} + H_{\rm vac} + H_{\rm SC}, \tag{23}$$

where $H_{\rm IGP}$, $H_{\rm vac}$, and $H_{\rm SC}$ correspond, respectively, to the Hamiltonians of the IGP of BLG, two edge regions placed at the top and bottom interfaces to vacuum accounting for the expected formation of trivial edge channels, and the two s-wave superconducting leads at $x = 0$ and $x = L$ with phase difference ϕ treated in mean-field. Setting the origin at the left bottom site of the NS interface, and using the same gauge for the vector potential as in the four-site model, the previous terms can be written as:

$$H_{\rm IGP} = \sum_{i,\sigma} (2t - \mu_N)\, c^\dagger_{i,\sigma} c_{i,\sigma} + \sum_{\langle i,j\rangle,\sigma} \left(t \exp\left\{ -i\pi \frac{\Phi}{\Phi_0} \frac{x_i(y_j - y_i)}{LW} \right\} c^\dagger_{i\sigma} c_{j\sigma} + \text{h.c.} \right), \tag{24}$$

$$H_{\rm vac} = \sum_{i,\sigma} (2t - \mu_{\rm vac})\, c^\dagger_{i,\sigma} c_{i,\sigma} + \sum_{\langle i,j\rangle,\sigma} \left(t c^\dagger_{i\sigma} c_{j\sigma} + \text{h.c.} \right), \tag{25}$$

$$H_{\rm SC} = H^L_{\rm SC} + H^R_{\rm SC}, \tag{26}$$

$$H^L_{\rm SC} = \sum_{i\in L,\sigma} (2t - \mu_{\rm SC})\, c^\dagger_{i,\sigma} c_{i,\sigma} - \sum_{i\in L} \left(\Delta c^\dagger_{i\uparrow} c^\dagger_{i\downarrow} + \text{h.c} \right), \tag{27}$$

$$H^R_{\rm SC} = \sum_{i\in R,\sigma} (2t - \mu_{\rm SC})\, c^\dagger_{i,\sigma} c_{i,\sigma} - \sum_{i\in R} \left(\Delta e^{i\phi} \exp\left\{ -i2\pi \frac{\Phi}{\Phi_0} \frac{x_i y_i}{LW} \right\} c^\dagger_{i\uparrow} c^\dagger_{i\downarrow} + \text{h.c} \right), \tag{28}$$

where $c^\dagger_{i\sigma}$ creates at electron with spin σ at site i, $\mu_{\rm N}$, $\mu_{\rm vac}$, and $\mu_{\rm SC}$ are the chemical potentials at the helical, vacuum-edge, and superconducting regions, respectively, and $t = \frac{\hbar^2}{2ma_0^2}$ where a_0 is the lattice constant and m the electron mass. Note that in this Section Δ denotes

the parent superconducting pairing amplitude (instead of the induced one used in the four-site model and the main text). Channels in the vacuum-edge region are assumed to be decoupled from each other and ballistic. Finally, the helical region corresponding to the IGP only "sees" the superconductors through its coupling to the vacuum-edge region [see Fig. 4(c) of the main text], therefore the coupling Hamiltonian reads:

$$H_{\text{coupling}} = H_{\text{IGP-vac}} + H_{\text{vac-SC}}, \tag{29}$$

with

$$H_{\text{IGP-vac}} = \sum_{\langle i,j\rangle,\sigma}^{\{i,j\}\in\{\text{IGP, vac}\}} \left(\tau_{\text{IGP-vac}} c^{\dagger}_{i\sigma} c_{j\sigma} + \text{h.c.}\right), \tag{30}$$

and

$$H_{\text{vac-SC}} = \sum_{\langle i,j\rangle,\sigma}^{\{i,j\}\in\{\text{BLG, vac}\}} \left(\tau_{\text{NS}} c^{\dagger}_{i\sigma} c_{j\sigma} + \text{h.c.}\right), \tag{31}$$

where $\tau_{\text{IGP-vac}} \leq t$ and $\tau_{NS} \leq t$ are the hopping amplitudes between sites at the IGP and thee vacuum edge and between the vacuum edge and the superconductor, respectively. Their magnitudes control the inter-edge coupling rate and transparency at the NS interfaces.

The $I_c(\Phi)$ shown in Fig. 4(d,e) of the main text is computed using Eqs. (2) and (1) and the spectrum of Eq. (22).

13. EVEN-ODD EFFECT: SIMPLE MODEL

The residual bulk conductance indeed leads to a large central supercurrent peak. However this additional effect doesn't completely mask the even-odd interference originating from the supercurrent carried by helical edge states. At a simplified level, we can understand its impact on the standard Fraunhofer and SQUID patterns as follows. As proposed by de Vries *et al.*[9], the even-odd effect can be qualitatively modelled by a flux-independent supercurrent offset f (that encodes the coupling between the two vacuum edges) in the SQI, i. e. $I_c(\Phi) \propto |\mathcal{I}(\Phi) + f|$. For a homogeneous JJ with uniform current flow $\mathcal{I}(\Phi) = \sin(\pi\Phi/\Phi_0)/(\pi\Phi/\Phi_0)$, while in the SQUID regime $\mathcal{I}(\Phi) = \cos(\pi\Phi/\Phi_0)$ [see Eq. (5) in the supplementary material]. We show the resulting SQI patterns for $f = 0$ (without the edge states or uncoupled edges) and $f = 0.05$ (finite coupling between the edge states) in Fig. S15. We see that in the homogeneous JJ case, the even-odd modulation produced by the edge modes is non-periodic, but still clearly visible, as in our experiment (Fig. 5 of the main

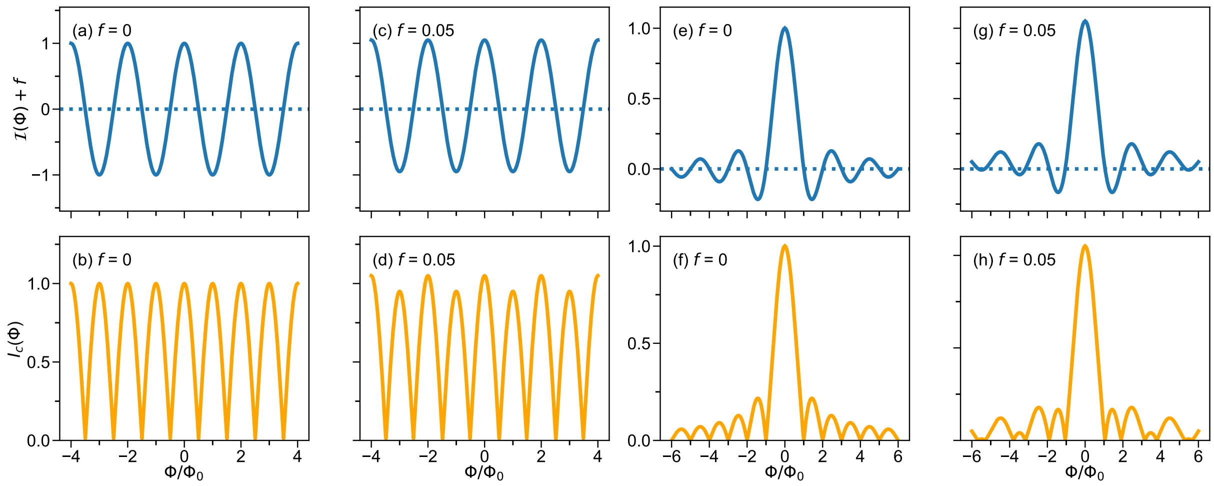

FIG. S15. The SQI function $\mathcal{I}(\Phi)+f$ for a symmetric SQUID with uncoupled (a) and coupled edges (c). (b, d) Lower panels show the corresponding critical currents for $I_{c,0} = 1$. The SQI function for a homogeneous JJ with uncoupled (e) and coupled edges (g). The corresponding critical currents for $I_{c,0} = 1$ are shown in the panels (f) and (h).

text). Similar SQI patterns with both large central peak and even-odd modulation have been reported for InSb[9] and HgTe[10] JJs previously.

SUPPLEMENTARY REFERENCES

[1] Y. Harada, S. Jensen, T. Akazaki, and H. Takayanagi, Physica C: Superconductivity **367**, 229 (2002).

[2] J. Paajaste, M. Amado, S. Roddaro, F. Bergeret, D. Ercolani, L. Sorba, and F. Giazotto, Nano Lett. **15**, 1803 (2015).

[3] H. J. Suominen, J. Danon, M. Kjaergaard, K. Flensberg, J. Shabani, C. J. Palmstrøm, F. Nichele, and C. M. Marcus, Phys. Rev. B **95**, 035307 (2017).

[4] J. Kroll, F. Borsoi, K. van der Enden, W. Uilhoorn, D. de Jong, M. Quintero-Pérez, D. van Woerkom, A. Bruno, S. Plissard, D. Car, E. Bakkers, M. Cassidy, and L. Kouwenhoven, Phys. Rev. Appl. **11**, 064053 (2019).

[5] M. T. Allen, O. Shtanko, I. C. Fulga, A. Akhmerov, K. Watanabe, T. Taniguchi, P. Jarillo-Herrero, L. S. Levitov, and A. Yacoby, Nat. Phys. **12**, 128 (2016).

[6] M. Zhu, A. Kretinin, M. D. Thompson, D. Bandurin, S. Hu, G. Yu, J. Birkbeck, A. Mishchenko, I. J. Vera-Marun, K. Watanabe, *et al.*, Nat. Commun. **8**, 1 (2017).

[7] B. Baxevanis, V. P. Ostroukh, and C. W. J. Beenakker, Phys. Rev. B **91**, 041409 (2015).

[8] H. Y. Ling and B. Kain, Phys. Rev. A **104**, 013305 (2021).

[9] F. K. de Vries, M. L. Sol, S. Gazibegovic, R. L. M. o. h. Veld, S. C. Balk, D. Car, E. P. A. M. Bakkers, L. P. Kouwenhoven, and J. Shen, Phys. Rev. Res. **1**, 032031 (2019).

[10] E. Bocquillon, R. S. Deacon, J. Wiedenmann, P. Leubner, T. M. Klapwijk, C. Brüne, K. Ishibashi, H. Buhmann, and L. W. Molenkamp, Nat. Nanotechnol. **12**, 137 (2017).
